# Supplementary material for: Descriptions of four new species of Minyomerus Horn, 1876 sec. Jansen & Franz, 2018 (Coleoptera: Curculionidae), with notes on their distribution and phylogeny
Source: PeerJ. 2018 Oct 16;6:e5633. doi: 10.7717/peerj.5633 (PMC6195114; doi:10.7717/peerj.5633)
Supplement: Supplemental Information 2 [file peerj-06-5633-s002.pdf]

Descriptions of four new species of *Minyomerus* Horn, 1876 sec. Jansen & Franz, 2018 (Coleoptera: Curculionidae), with notes on their distribution and phylogeny

Supplemental Information SI1

Explanation of the RCC–5 alignment approach

All alignments were generated with the Euler/X reasoner toolkit (Chen et al. 2014), available at https://github.com/EulerProject/. To generate the input constraints, the more recent classification or phylogeny (T_2 – 2018) was represent first, whereas the preceding classification or phylogenetic (T_1 – 2015) was represent second.

The rank-only classification alignment (**SI2**) is straightforward, except for two particular conventions.

First, this and the other two alignments are *intensional* in their configuration. This means that they are intended to express congruence among higher-level parent concepts – such as *Minyomerus* [JF2018] and *Minyomerus* [JF2015] – in spite of non-congruently sampled child concepts. In this case, because the present revision adds four species-level concepts to *Minyomerus* [JF2018] in comparison to *Minyomerus* [JF2015], the genus concept-level congruence *Minyomerus* [JF2018] == [JF2015] is only attainable (i.e., logically consistent) if the coverage constraint is relaxed for 2015.Minyomerus (now using the toolkit input syntax), by adding 2015.nc_Minyomerus.

Second, as much as appeared sensible, we avoid authoring "redundant" taxonomic or clade concept labels in the current (2018) revision. In particular, given our assessment that none of the 17 species-level concepts placed in *Minyomerus* [JF2015] are in question for this current revision, no new (2018) taxonomic concept labels were coined for these. This means, then, that parent coverage must be relaxed reciprocally: (1) in parent concepts sec. Jansen & Franz (2015) to accommodate species-level concepts not known and hence not sampled therein, and also (2) in parent concepts sec. Jansen & Franz (2018) that intentionally "lack" all species-level concepts that are already accounted for, so to speak, in the earlier revision's input.

These conventions explain the input data file for SI2:

```
taxonomy 2018 JansenFranz
(Minyomerus [4 new species-level concepts] nc_Minyomerus)

taxonomy 2015 JansenFranz
(Minyomerus [17 existing species-level concepts] nc_Minyomerus)

articulation 2018_2015 JansenFranz_JansenFranz
[2018.Minyomerus = 2015.Minyomerus]
[2018.nc_Minyomerus > {all 17 species-level concepts of taxonomy 2015}]
[{all 4 species-level concepts of taxonomy 2018} < 2015.Minyomerus]
```

The standard toolkit commands (status: April, 2018) used to obtain the alignments are:

```
euler2 align [filename] -r clingo   (infers the alignment)
euler2 show iv                      (generates input visualization)
euler2 show pw                      (generates alignment visualization)
```

The multi-phylogeny alignments (SI3 and SI4) constitutes two toolkit runs based on the same data input. Clade concept labels are now consistently generated by combining the *alphabetically first-appearing epithet* from (anywhere in) the two sister concepts, or concept groups, bifurcating from the corresponding node. The two species-level taxonomic concept labels are then ordered – again – alphabetically to generate the clade concept label.

So, for instance, the label for the clade concept {(*M. ampullaceus,* (*M. aeriballux, M. reburrus*))} sec. Jansen & Franz (2018) becomes "*M. aeriballux–M. ampullaceus* clade [JF2018]" in the main revision text, or "2018.Minyomerus_aeriballux_Minyomerus_ampullaceus_Clade" in the reasoner toolkit input.

The multi-phylogeny alignment is further "pruned" not just to avoid redundant species-level taxonomic concept labels, but to apply this convention also to reduce – though not fully eliminate – redundant clade-level concepts. In order words, the alignment visualizations nearly maximize the numbers of green rectangular (2018) and yellow octagonal (2015) regions at the leaves and lower node levels, which would be grey/rounded corner rectangles otherwise.

In particular, the alignment reflects the following assertions regarding the four species-level concepts described in 2018:

1. ***Minyomerus ampullaceus* [JF2018]** is intensionally subsumed under the clade concept of *M. aeriballux–M. bulbifrons* [JF2015].

2. ***Minyomerus franko* [JF2018]** is intensionally subsumed under the clade concept of *M. caseyi–M. trisetosus* [JF2015].

3. ***Minyomerus sculptilis* [JF2018]** is intensionally subsumed under the clade concept of *Minyomerus* [JF2015].

4. ***Minyomerus tylotos* [JF2018]** is intensionally subsumed under the clade concept of *M. aeriuallux–M. microps* [JF2015].

This convention requires nine instances of relaxing the coverage constraint; 5 in the 2018 phylogeny and 4 in the 2015 phylogeny. It also requires a sufficiently large number of higher-level RCC–5 input articulations to constrain the set of possible worlds as intended, viz., reflective of shared property-based (synapomorphy, homoplasy) circumscriptions of the 2018/2015 clade concepts. Examples of such articulations are:

```
articulation 2018-2015 JansenFranz_JansenFranz
[2018.Minyomerus_ampullaceus =
2015.nc_Minyomerus_aeriballux_Minyomerus_bulbifrons_Clade]

[2018.Minyomerus_franko =
2015.nc_Minyomerus_caseyi_Minyomerus_trisetosus_Clade]

[2018.Minyomerus_sculptilis = 2015.nc_Minyomerus]

[2018.Minyomerus_tylotos <
2015.Minyomerus_aeriballux_Minyomerus_microps_Clade]

[2018.Minyomerus_aeriballux_Minyomerus_cracens_Clade =
2015.Minyomerus_aeriballux_Minyomerus_bulbifrons_Clade]
```

```
[2018.Minyomerus_caseyi_Minyomerus_franko_Clade =
2015.Minyomerus_caseyi_Minyomerus_trisetosus_Clade]
```

The toolkit run for the **SI3** data file set uses the above commands. However, the additional run for the **SI4** data file set uses the modified commands:

```
euler2 align [filename] -e mncb -r clingo (uses split-concept resolution)
euler2 show iv                            (generates input visualization)
euler2 show pw --hideoverlaps             (generates alignment visualization)
```

The GraphViz output data files that generate the alignment visualizations were edited for SI3 and SI4 to show a "bottom-top" orientation (rankdir=BT), when "left-right" (rankdir=LR) is the default.

Lastly, for each set of **SI2** to **SI4** reasoner toolkit input/output, the following data files are provided.

A. The reasoner input constraints for the particular alignment, with alignment metadata and run commands. File format: .txt.

B. The input visualization for the alignment. File format: .pdf.

C. The set of *Maximally Informative Relations* (MIR) inferred for the alignment; see Franz et al. (2016a, 2016b). File format: .csv.

D. The alignment visualization for the alignment. File format: .pdf.
