# Supplementary figures and images for: Descriptions of four new species of Minyomerus Horn, 1876 sec. Jansen & Franz, 2018 (Coleoptera: Curculionidae), with notes on their distribution and phylogeny
Source: PeerJ. 2018 Oct 16;6:e5633. doi: 10.7717/peerj.5633 (PMC6195114; doi:10.7717/peerj.5633)

| Nodes         |    |
|---------------|----|
| 2015          | 19 |
| 2018          | 6  |
| Edges         |    |
| is_a (2015)   | 18 |
| is_a (2018)   | 5  |
| articulations | 22 |

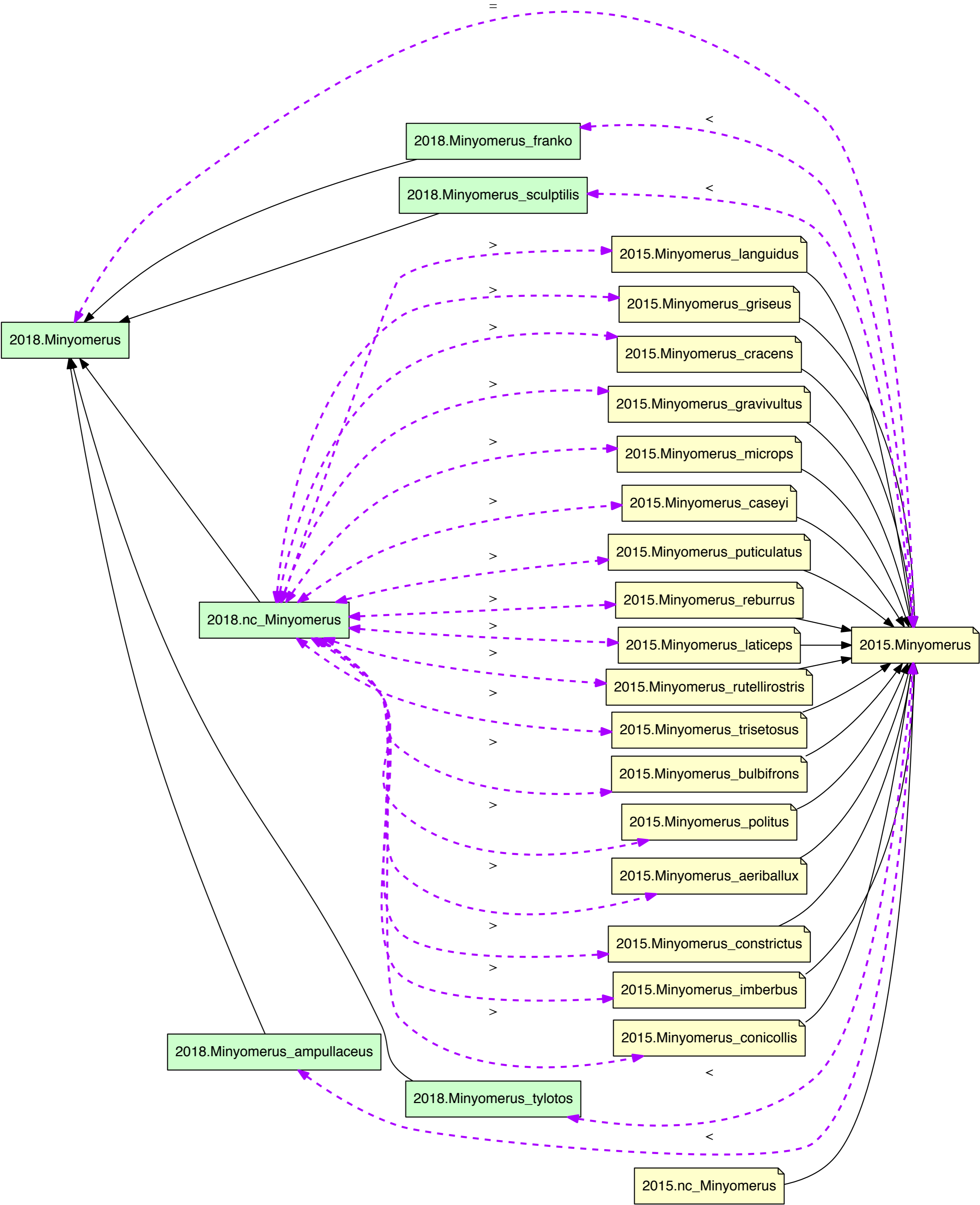

Supplement: Supplemental Information 4 — Input visualization for the SI2A input. [file peerj-06-5633-s004.pdf]

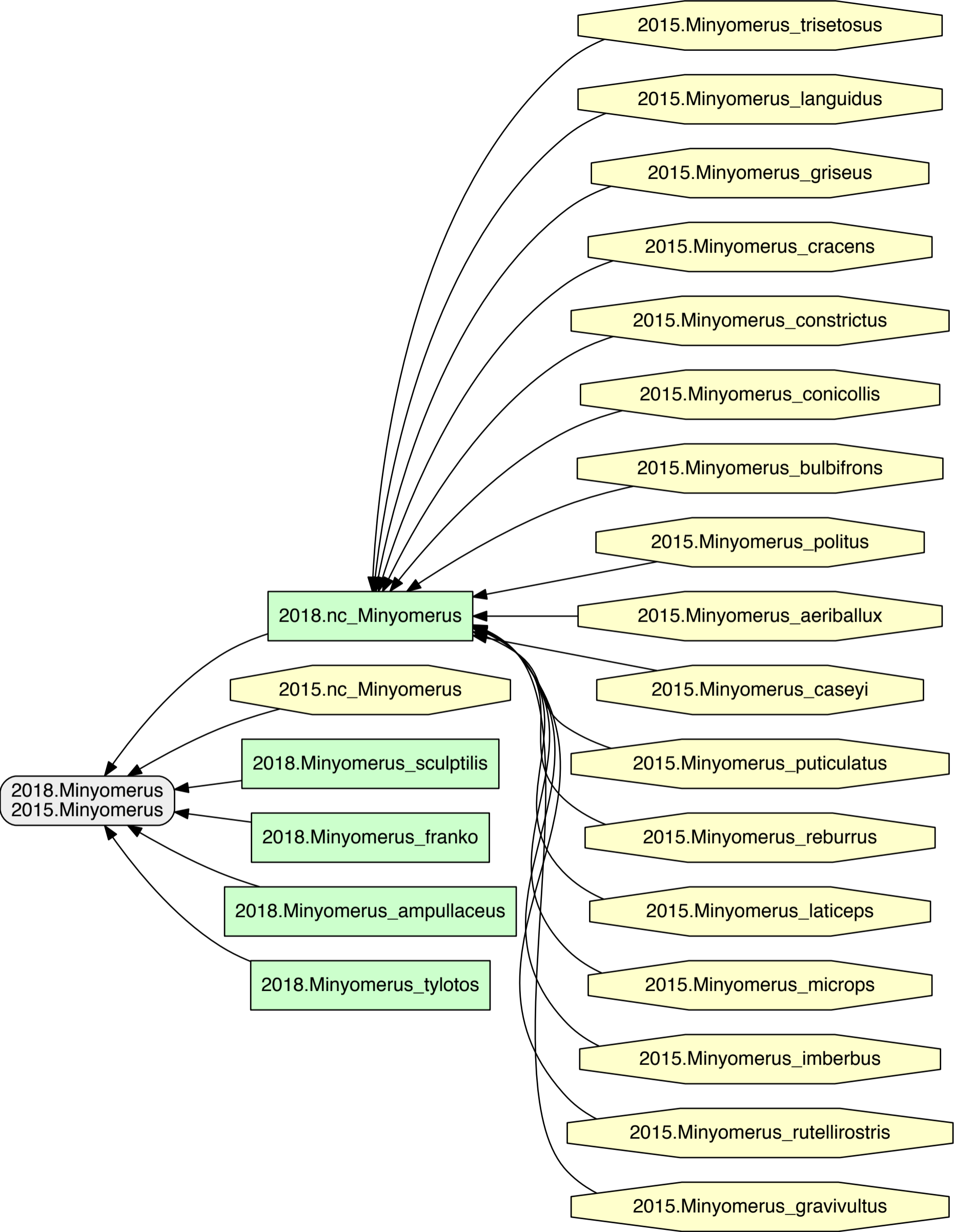

| Nodes        |    |
|--------------|----|
| 2015         | 18 |
| congruent    | 1  |
| 2018         | 5  |
| Edges        |    |
| is_a (input) | 23 |

Supplement: Supplemental Information 6 — Alignment visualization for the SI2A input. [file peerj-06-5633-s006.pdf]

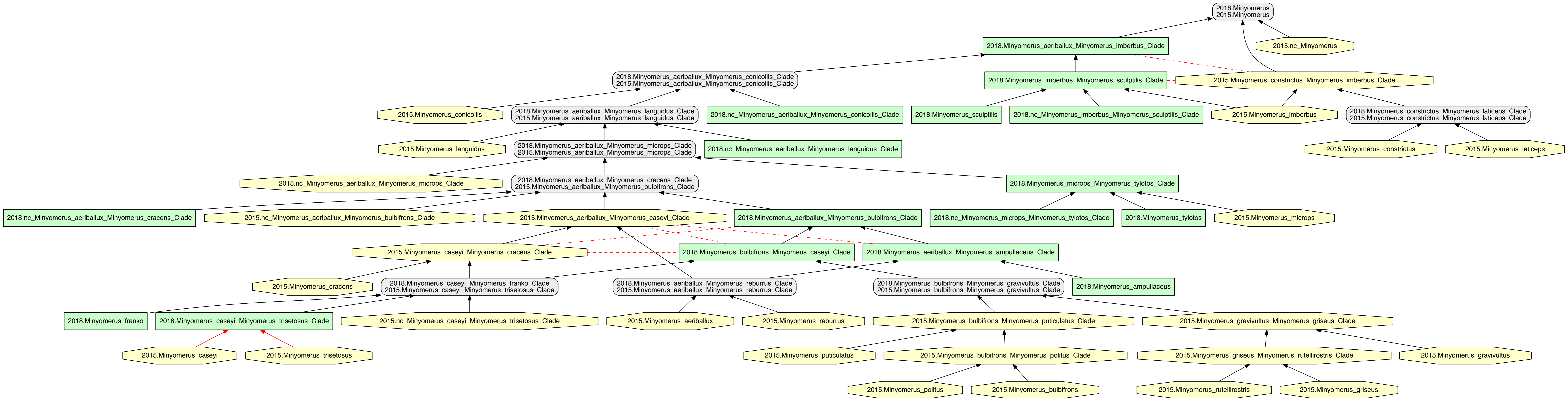

| Nodes               |    |
|---------------------|----|
| 2015                | 28 |
| congruent           | 9  |
| 2018                | 16 |
| Edges               |    |
| is_a (inferred)     | 2  |
| is_a (input)        | 53 |
| overlaps (inferred) | 7  |

Supplement: Supplemental Information 10 — Alignment visualization for the SI3A input. [file peerj-06-5633-s010.pdf]
