# Supplementary material for: Descriptions of four new species of Minyomerus Horn, 1876 sec. Jansen & Franz, 2018 (Coleoptera: Curculionidae), with notes on their distribution and phylogeny
Source: PeerJ. 2018 Oct 16;6:e5633. doi: 10.7717/peerj.5633 (PMC6195114; doi:10.7717/peerj.5633)
Supplement: Supplemental Information 8 — Input visualization for the SI3A input. [file peerj-06-5633-s008.pdf]

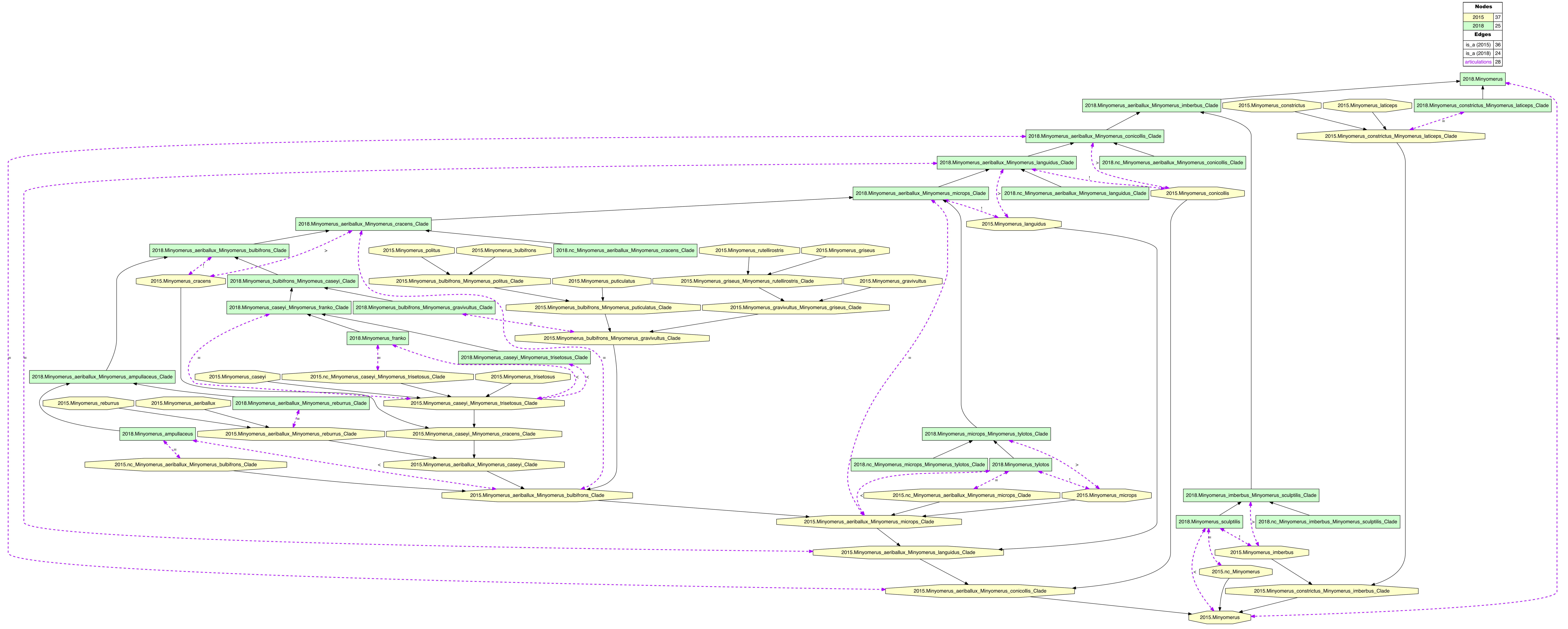
Nodes
2015 37
2018 25
Edges
is_a (2015) 36
is_a (2018) 24
articulations 28
2018.Minyomerus
2018.Minyomerus_aeriballux_Minyomerus_imberbus_Clade
2015.Minyomerus_constrictus
2015.Minyomerus_laticeps
2018.Minyomerus_constrictus_Minyomerus_laticeps_Clade
2018.Minyomerus_aeriballux_Minyomerus_conicollis_Clade
2015.Minyomerus_constrictus_Minyomerus_laticeps_Clade
2018.Minyomerus_aeriballux_Minyomerus_languidus_Clade
2018.nc_Minyomerus_aeriballux_Minyomerus_conicollis_Clade
2018.Minyomerus_aeriballux_Minyomerus_microps_Clade
2018.nc_Minyomerus_aeriballux_Minyomerus_languidus_Clade
2015.Minyomerus_conicollis
2015.Minyomerus_languidus
2018.Minyomerus_aeriballux_Minyomerus_cracens_Clade
2018.Minyomerus_aeriballux_Minyomerus_bulbifrons_Clade
2015.Minyomerus_politus
2015.Minyomerus_bulbifrons
2018.nc_Minyomerus_aeriballux_Minyomerus_cracens_Clade
2015.Minyomerus_rutellirostris
2015.Minyomerus_griseus
2015.Minyomerus_cracens
2018.Minyomerus_bulbifrons_Minyomerus_caseyi_Clade
2015.Minyomerus_bulbifrons_Minyomerus_politus_Clade
2015.Minyomerus_puticulatus
2015.Minyomerus_griseus_Minyomerus_rutellirostris_Clade
2015.Minyomerus_gravivultus
2018.Minyomerus_caseyi_Minyomerus_franko_Clade
2018.Minyomerus_bulbifrons_Minyomerus_gravivultus_Clade
2015.Minyomerus_bulbifrons_Minyomerus_puticulatus_Clade
2015.Minyomerus_gravivultus_Minyomerus_griseus_Clade
2018.Minyomerus_franko
2015.Minyomerus_bulbifrons_Minyomerus_gravivultus_Clade
2018.Minyomerus_caseyi_Minyomerus_trisetosus_Clade
2018.Minyomerus_aeriballux_Minyomerus_ampullaceus_Clade
2015.Minyomerus_caseyi
2015.nc_Minyomerus_caseyi_Minyomerus_trisetosus_Clade
2015.Minyomerus_trisetosus
2015.Minyomerus_reburrus
2015.Minyomerus_aeriballux
2018.Minyomerus_aeriballux_Minyomerus_reburrus_Clade
2015.Minyomerus_caseyi_Minyomerus_trisetosus_Clade
2018.Minyomerus_ampullaceus
2015.Minyomerus_aeriballux_Minyomerus_reburrus_Clade
2015.Minyomerus_caseyi_Minyomerus_cracens_Clade
2018.Minyomerus_microps_Minyomerus_tylotos_Clade
2015.nc_Minyomerus_aeriballux_Minyomerus_bulbifrons_Clade
2015.Minyomerus_aeriballux_Minyomerus_caseyi_Clade
2018.nc_Minyomerus_microps_Minyomerus_tylotos_Clade
2018.Minyomerus_tylotos
2015.Minyomerus_aeriballux_Minyomerus_bulbifrons_Clade
2015.nc_Minyomerus_aeriballux_Minyomerus_microps_Clade
2015.Minyomerus_microps
2018.Minyomerus_imberbus_Minyomerus_sculptilis_Clade
2015.Minyomerus_aeriballux_Minyomerus_microps_Clade
2018.Minyomerus_sculptilis
2018.nc_Minyomerus_imberbus_Minyomerus_sculptilis_Clade
2015.Minyomerus_aeriballux_Minyomerus_languidus_Clade
2015.Minyomerus_imberbus
2015.nc_Minyomerus
2015.Minyomerus_aeriballux_Minyomerus_conicollis_Clade
2015.Minyomerus_constrictus_Minyomerus_imberbus_Clade
2015.Minyomerus
